# Entomological Characteristics of Malaria Transmission across Benin: An Essential Element for Improved Deployment of Vector Control Interventions

**DOI:** 10.3390/insects14010052

**Published:** 2023-01-05

**Authors:** Tatchémè Filémon Tokponnon, Razaki Ossè, Germain Gil Padonou, Cyriaque Dossou Affoukou, Aboubakar Sidick, Wilfried Sewade, Arsène Fassinou, Côme Z. Koukpo, Bruno Akinro, Louisa A. Messenger, Mariam Okê, Alexis Tchévoédé, Aurore Ogouyemi-Hounto, Dorothée Kinde Gazard, Martin Akogbeto

**Affiliations:** 1 Ministère de la Santé, Cotonou 08 BP 882, Benin; 2 Cotonou Entomological Research Center, Cotonou 06 BP 2604, Benin; 3 Centre Béninois de la Recherche Scientifique et de l’Innovation (CBRSI), Agbondjèdo, Étoile Rouge, Cotonou 03 BP 1665, Benin; 4 Ecole Polytechnique d’Abomey-Calavi, Université d’Abomey-Calavi, Cotonou 01 BP 2009, Benin; 5 National Malaria Control Program, Cotonou 01 BP 882, Benin; 6 Department of Disease Control, London School of Hygiene & Tropical Medicine, London WC1E 7HT, UK; 7 Department of Environmental and Occupational Health, School of Public Health, University of Nevada, Las Vegas, NV 89154, USA; 8 Parasitology-Mycologie Research Unit, Faculté des Sciences de la Santé, University of Abomey-Calavi, Cotonou 01 BP 188, Benin

**Keywords:** heterogeneous transmission, malaria, *Anopheles gambiae s.l.*, vector control, intervention deployment, Benin, West Africa

## Abstract

**Simple Summary:**

With limited resources to properly perform geographic surveillance of malaria vectors, Benin, through its National Malaria Control Program (NMCP), conducted a study on vector dynamics and transmission across the country’s departments in order to generate an entomological transmission profile. Per department, 2 communes (i.e., 24 across twelve departments) were chosen for this study, taking into account geographical and ecological diversity. We selected two villages per commune and four households (HH) per village to collect mosquitoes using human landing catches (HLCs). In each HH, an indoor and outdoor HLC session took place between 7 p.m. and 7 a.m. on two consecutive nights between July and September 2017. Captured *Anopheles* were identified and ovaries were dissected for parity. Heads and thoraxes were tested for *Plasmodium falciparum* sporozoites by ELISA, and entomological inoculation rate (EIR) was calculated. Analyses revealed low, medium, and high transmission communes distributed throughout the country. Heterogeneity in malaria transmission in Benin was observed, which is not evident when assessing entomological surveillance from sentinel sites alone. The NMCP will use the study findings in the future to stratify and plan vector control interventions in high transmission districts to better protect the most at-risk populations.

**Abstract:**

Entomological surveillance in Benin has historically been limited to zones where indoor residual spraying was performed or where long-standing sentinel surveillance sites existed. However, there are significant country-wide gaps in entomological knowledge. The National Malaria Control Program (NMCP) assessed population dynamics of *Anopheles* vectors and malaria transmission in each of Benin’s 12 departments to create an entomological risk profile. Two communes per department (24/77 communes) were chosen to reflect diverse geographies, ecologies and malaria prevalence. Two villages per commune were selected from which four households (HH) per village were used for human landing catches (HLCs). In each HH, an indoor and outdoor HLC occurred between 7 p.m. and 7 a.m. on two consecutive nights between July–September 2017. Captured *Anopheles* were identified, and ovaries were dissected to determine parous rate. Heads and thoraces were tested for *Plasmodium falciparum* sporozoites by ELISA. The Entomological Inoculation Rate (EIR) was calculated as the product of mosquito bite rate and sporozoite index. Bite rates from *An. gambiae s.l.*, the primary vector species complex, differed considerably between communes; average sporozoite infection index was 3.5%. The EIR ranged from 0.02 infectious bites (ib) per human per night in the departments of Ouémé and Plateau to 1.66 ib/human/night in Collines. Based on transmission risk scales, Avrankou, Sakété and Nikki are areas of low transmission (0 < EIR < 3 ib/human/year), Adjarra, Adja Ouèrè, Zè, Toffo, Bopa, Pehunco, Pèrèrè and Kandi are of medium transmission (3 < EIR < 30 ib/human/year), and the other remaining districts are high transmission (EIR > 30 ib/human/year). The heterogeneous and diverse nature of malaria transmission in Benin was not readily apparent when only assessing entomological surveillance from sentinel sites. Prospectively, the NMCP will use study results to stratify and deploy targeted vector control interventions in districts with high EIRs to better protect populations most at-risk.

## 1. Introduction

Among vector-borne diseases, malaria remains one of the most important parasitic infections worldwide [1]. In sub-Saharan Africa (SSA), malaria plays a major role in the poor economic development of certain endemic regions where transmission is very intense [2]. In 2021, the World Malaria Report estimated that there were 241 million cases of malaria and 627,000 deaths from malaria worldwide in 2020 [3]. The proliferation of mosquitoes is promoted not only by ecological changes due to human activities (including, deforestation, public works, construction of dams, rice paddies and irrigation) but also by environmental parameters (rainfall, temperature, and relative humidity), which play a fundamental role in moderating the level of transmission and the epidemiology of disease [4,5,6]. In intertropical Africa, malaria transmission is very heterogeneous due to eco-climatic variations [7]. Currently, five parasitic species of the *Plasmodium* genus have been identified as responsible for malaria infection in humans [8]. Among them *Plasmodium falciparum* remains the most virulent species causing deadly forms of malaria [9]. The *Plasmodium* species responsible for human malaria are mainly transmitted by primary vector species, such as *Anopheles gambiae* sensu lato (s.l.), *Anopheles funestus* group and *Anopheles nili* group [10,11], which are characterized by different feeding and resting behaviors. Sympatric overlap of these different species complexes in an area represents a significant challenge for malaria control programs.

Reductions in malaria morbidity have been observed over the past decade but have coincided with a massive scale-up of prevention measures of which vector control is an important component [11,12]. Residual malaria transmission, defined by the World Health Organization as the “persistence of parasite transmission, even with good access and the use of well-implemented insecticide-treated nets (ITNs) or indoor residual spraying (IRS), as well as in situations where ITNs or IRS use is impractical,” is a critical challenge to malaria control and elimination efforts [13,14].

In Benin, malaria has for several years been the leading infectious disease and the primary reason for seeking care in health facilities. According to the health statistics directory generated by the Ministry of Health’s National Health Information and Management System (SNIGS) for 2019, malaria accounted for 45.5% and 48.8% of the reasons for consultation and hospitalization among the general population and among children under five years of age, respectively. The incidence of malaria in 2019 was 21.2% in the general population [15] and the parasitic prevalence in children under 5 years of age was 37.3% [16]. Despite efforts to combat this disease, morbidity and mortality have not decreased much in recent years in Benin. The limited availability of epidemiologically reliable baseline data renders it difficult to make evidence-based decisions when choosing control strategies. While data exist on the entomological profile of malaria transmission in Benin, they are sparse and do not cover the entire country [17,18,19,20]. However, malaria is endemic throughout the country with several interventions being deployed in the field, including case management, promotion of long-lasting insecticidal nets (LLINs), IRS, behavioral and social change communication strategies (BCCS), greater availability of effective and affordable artemisinin-based combination therapy (ACT), intermittent preventive treatment for pregnant women (IPTp) and seasonal chemoprophylaxis of malaria (SMC); as well as sanitation measures for the living environment, which are poorly implemented [21].

In Benin, over the past two decades, the roles of *An. gambiae s.l.* and *An. funestus* group in the transmission of *P. falciparum* in several regions of the country have been studied by multiple authors [17,18,22,23,24,25,26,27,28]. Unfortunately, the emergence and spread of resistance to pyrethroid insecticides in many mosquito populations has made control difficult [29]. African malaria vector populations are becoming increasingly resistant to almost all insecticides used for malaria prevention [30,31,32].

Within this framework, the National Malaria Control Program (NMCP) in Benin has planned, to establish and periodically update an entomological profile of malaria transmission to improve disease prevention preparedness. It is necessary to assess in the same period the intensity of malaria transmission across different departments of the country and to identify the responsible vector species [21]. The objective of this study was to carry out an entomological survey of malaria transmission in all departments of the country. Specifically in a single transmission period, this study (i) assessed Culicidae diversity and *Anopheles* populations involved in malaria transmission in all departments; (ii) determined biting rate, longevity, level of malaria transmission and insecticide resistance mechanisms present in *Anopheles* vectors in all departments; and (iii) mapped the burden of malaria transmission in all departments to better deploy future vector control interventions.

## 2. Materials and Methods

### 2.1. Study Sites

**Figure 1:**
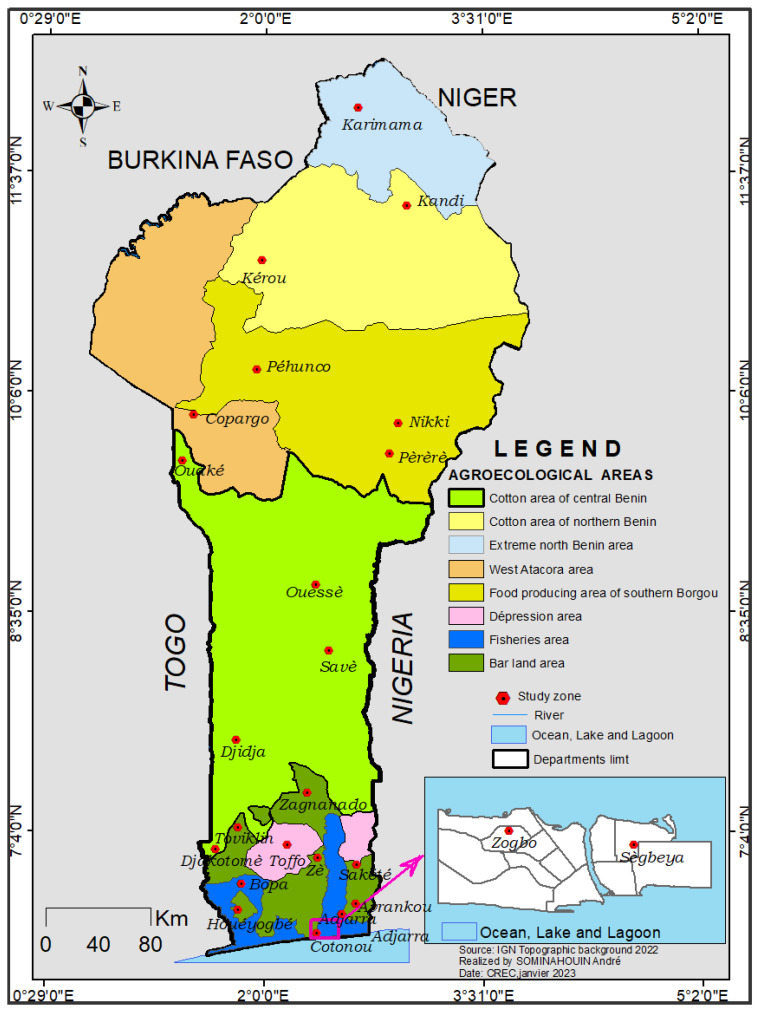
Map showing the study sites in the 12 departments presented according to the 8 eco-agricultural zones.

This study was carried out in all departments of Benin from July to December 2017. Out of a total of 77 communes, 24 were selected, with two communes per department. Twenty-four communes were chosen to encompass the eight agro-ecological zones, classified based on climatic and agro-pedological parameters, cropping systems, population density, plant cover and certain logistical constraints (Figure 1). Karimama commune in zone 1 marks the northern limit of Benin by the presence of the Niger River and benefits from its three tributaries, Mekrou, Alibori and Sota. It is an area which contains in its major part the W National Park of Niger forest reserve. Its climate is of the Sudano-Sahelian type and includes the communes of Malanville and Karimama. Temperatures are excessive and reach 40 °C in the shade during the dry season.

The communes of Kandi and Kérou in zone 2 are in the cotton cultivation area of northern Benin. This area is watered by the same tributaries of the Niger River as zone 1 and is influenced by the continental trade winds.

The communes of Pèrèrè, Pehunco and Nikki in zone 3 are characterized by a very high availability of agricultural land, which is an important factor in the socio-economic development of the region and a major asset for food safety. This area has a humid Sudanese climate marked by a rainy season from April to September and a dry season that lasts almost five months.

The communes of Copargo and Ouaké in zone 4 benefit from the presence of the Atacora Mountain Range, where temperatures are cooler, and thunderstorms are more frequent than in other areas. Rainfall levels vary from 800 to 1350 mm. The main watercourse is the Pendjari River with its tributaries.

The communes of Savè, Ouèssè and Djidja were chosen in zone 5. This area is the largest and contains all the department of the hills and part of some other departments, in particular Borgou, Donga, Couffo, Plateau and Zou. It is an area conducive to agriculture and is home to most of the “agricultural colonizers” from zone 4. It is watered by the Ouémé River and its tributaries (the Zou and Okpara).

The communes of Zè, Adjarra, Avrankou, Sakété, Houéyogbé, Zangnanado, Toviklin and Djakotomey are in zone 6. This zone is one of the most complex and is called the “bare ground zone” due to the characteristics of local soils. The climate is marked by two rainy seasons (March-July and October-November) and two dry seasons (December-February and August); rainfall levels range from 1000 to 1400 mm.

The communes of Toffo and Adja-Ouèrè in zone 7 are located in the Chukchi Depression. In Mono department there is the commune of Lalo, the Lama depression in the Atlantic and Zou contains the communes of Toffo and Zogbodomey, respectively, and Issaba in the Ouémé department has the communes of Adja-Ouèrè and Pobè. It is the smallest of the eight agro-ecological zones in terms of area. The climate is comparable to zone 6 but with a higher relative humidity of approximately 85%.

Zone 8 includes the communes of Bopa and Cotonou. One of the main characteristics of this area is the development of inland and coastal fishing, as a complement to plant and animal production. Geographically, it is the most southern and occupies the fluvio-lacustrine zone of the Atlantic, Mono, Ouémé and Zou departments [33].

### 2.2. Monitoring of Anopheles Population Dynamics, Vector Species and Malaria Transmission

Sampling of *Anopheles* Populations

Collections of mosquitoes were performed using both human landing catches (HLCs) and pyrethrum spray catches (PSCs) during the rainy season. Two communes in each department (24/77 communes) were identified for transmission monitoring. These communes were different from those selected for the bio-ecology and vector resistance study. In each commune, two villages, one central and one peripheral, were chosen to assess malaria transmission. HLCs were carried out in four households (HH) per village to collect mosquitoes for the monitoring of malaria transmission. Between 7 p.m.–7 a.m. for 2 consecutive nights between July–September 2017, 2 sessions occurred; captures were organized in each HH, indoors and outdoors, for a total of 16 capture nights per village and a total of 64 capture nights per department.

PSCs were performed to assess the behavior of *Anopheles* vectors. Early in the morning in each district, 10 bedrooms were surveyed (5 in the central village and 5 in the peripheral village) to collect all mosquitoes that entered the houses the night before. Aerosol bombs (Rambo^®^) containing 0.25% transfluthrin and 0.20% permethrin were sprayed in houses and white canvas was spread on the floor to collect fallen mosquitoes. This operation allowed us to estimate mosquito density and identify vector blood meal sources.

Mosquitoes caught by both methods were morphologically identified using a taxonomic key [34]. A subset of unfed *An. gambiae s.l.* females from HLCs were dissected to extract ovaries and to determine parous *An. gambiae s.l.* (mosquitoes that oviposited at least once) by observing the coiling degree of ovarian tracheoles [35].

### 2.3. Laboratory Analysis

To detect the presence of *P. falciparum* circumsporozoite protein (CSP) [36], 2875 head-thoraxes of *Anopheles* vectors, collected indoors and outdoors by HLCs, were crushed and analyzed by direct enzyme-linked immunosorbent assay (ELISA) thus allowing the determination of the sporozoite index (SI).

The legs, wings, and abdomens of these mosquitoes were used for DNA extraction to perform molecular species identification, using the protocols of Santomalazza et al. [37], Koekemoer et al. [38] and Kengne et al. [39] to identify sibling species of the *An. gambiae* complex, *An. funestus* group and *An. nili* group, respectively. In approximately 60 *An. gambiae s.l.* per department, the presence of L1014F kdr and G119S Ace-1 mutations were assessed following the methods of Martinez-Torres et al. [40] and Weill et al. [41], respectively. Blood meal origin was identified in blood-fed *Anopheles* mosquitoes collected by PSC, using an ELISA according to the method of Beier et al. [42] with human, cattle, sheep, chicken and pig antibodies.

### 2.4. Elaboration of the Entomological Transmission Profile and Collection of Malaria Incidence and Prevalence Data

Based on the model proposed by Robert in 2004 [43], we retained three categories of endemic areas in the country. The areas with an EIR less than 3 infectious bites/house/year was considered low transmission (0 < EIR < 3 infectious bites/house/year); the area with an EIR between 3 and 30 infectious bites/house/year was medium transmission (3 < EIR < 30 infectious bites/house/year); and high transmission areas had EIR greater than 30 infectious bites/house/year (EIR > 30 infectious bites/house/year).

For this purpose, the EIR of each commune was used to produce a geographical profile of malaria transmission across Benin.

To strengthen this transmission profile, malaria prevalence and incidence data from each commune collected through the SNIGS and the Demographic Health Survey (DHS) during the same year were linked to the EIR. The vector control interventions implemented in the study communes were LLINs distributed every three years in mass campaigns and through targeted distributions from health centers, except for the communes of Kandi, Copargo, and Ouaké, which also benefited from IRS in 2017.

### 2.5. Data Analysis

Study results were analyzed using the R Core Team software (Version 3.5.1-2018) and Excel spreadsheets. The human biting rate (HBR = number of collected vectors/number of humans/number of nights), infection rates (number of infected mosquitoes/total tested), EIR (EIR night = HBR × infection rate; EIR month = EIR night × 30) and parity rates (number of parous mosquitoes/total dissected) were calculated and compared between vector species from the same district, using Poisson tests.

Chi-squared tests were used to compare parity rates, infectivity, and allelic frequencies of L1014F kdr and G119S Ace-1 mutations, by species and by site. Tests for correlation between the EIR, malaria prevalence and incidence in the different communes were also performed.

## 3. Results

### 3.1. Mosquito Species Diversity and Molecular Identification of Related Species of the Anopheles Gambiae Complex

**Figure 2:**
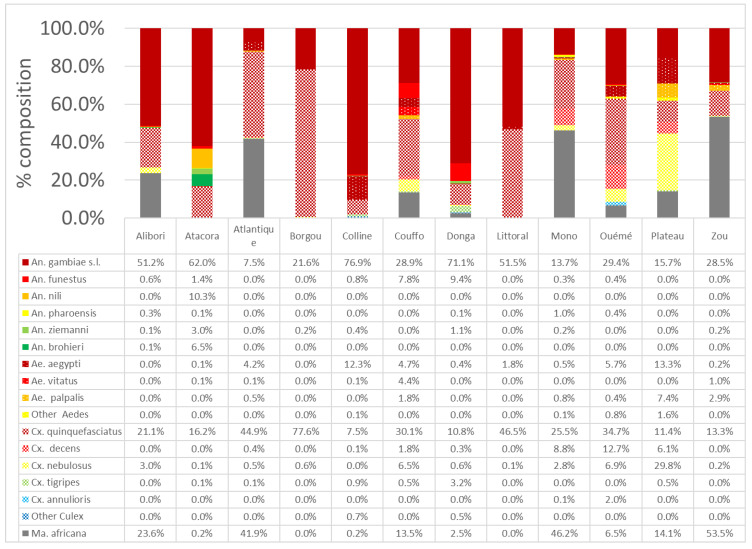
Culicidae diversity and abundance in the 12 departments of Benin.

Figure 2 shows the different species of mosquito collected in the departments of Benin during the study period. In total, 17,968 mosquitoes from 19 species (*Anopheles gambiae s.l, Anopheles funestus, Anopheles nili, Anopheles brohieri, Anopheles pharoensis, Anopheles ziemmani, Aedes aegypti, Ae. circumluteolus, Ae. vittatus, Ae. palpalis, Culex decens, Cx. nebulosus, Cx. quinquefasciatus, Cx. tigripes, Cx. fatigrans, Cx annulioris, Mansonia africana, Coquiletidia cristata* and *Uranotaenia bilineata*) were collected in the different communes. The departments of Ouémé and Mono were the most prolific with 13 species each, followed by the department of Atacora (12 species), then the departments of Donga, Couffo and Collines (11 species each). In the departments of Zou, Plateau, Atlantic, Alibori, Littoral and Borgou, the number of species recorded was 10, 10, 9, 8, 5 and 3, respectively.

In general, *An. gambiae s.l*, *Cx. quinquefasciatus*, *Ma. africana* and *Ae. aegypti* were the dominant species in the departments with abundances of 40.2%, 28.5%, 20.3% and 2.5%, respectively (Figure 2). Of all species collected by department, *An. gambiae s.l*. was the most abundant in the departments of Collines (76.8%), Donga (71.1%), Atacora (62.0%), Littoral (51.5%), Alibori (51.2%) and Borgou (21.5%). By comparison, in Ouémé, Atlantic and Couffo departments, *Cx. quinquefasciatus* was the dominant species with frequencies of 33.7%, 44.9% and 29.9%, respectively. *Ma. africana* predominated in the Zou (53.5%) and Mono (46.2%) departments, while *Cx. nebulosus* was abundant in the Plateau department (29.8%). *An. gambiae s.l.* represented 97.8% of malaria vectors; 1.2% were *An. funestus* group and 1.1% were *An. nili*.

### 3.2. Species and Allelic Frequency of L1014F kdr and G119S Ace-1 Mutations in An. gambiae s.l. Populations Involved in Malaria Transmission in Each Commune

**Figure 3:**
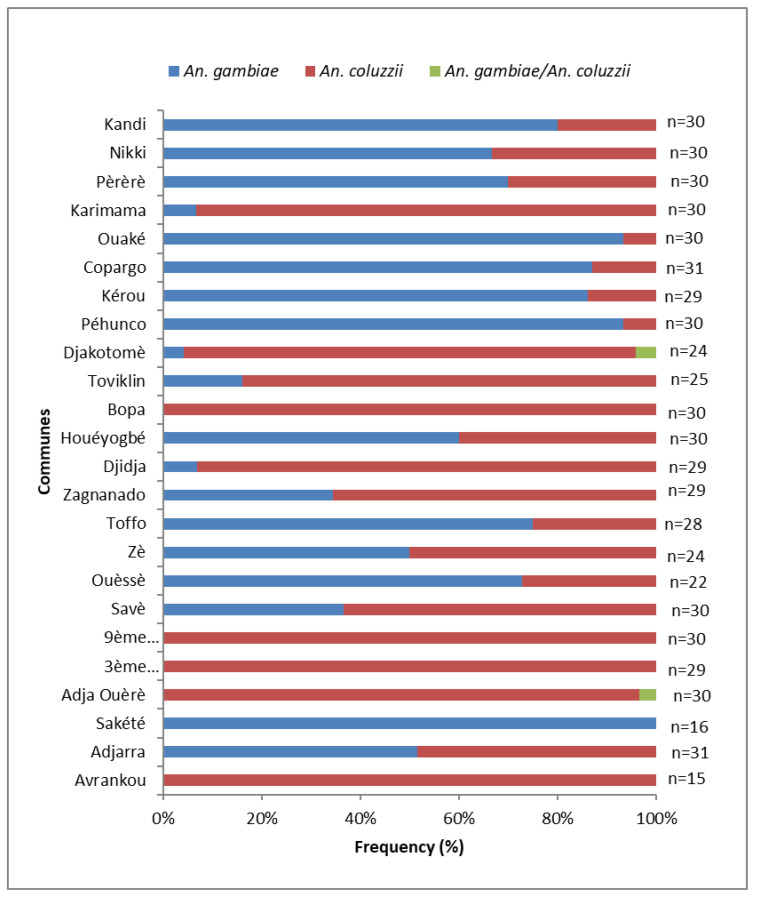
Distribution of related species of the *Anopheles gambiae* complex in the study communes.

Mosquitoes from each department were analyzed by PCR to identify the species present within the *An. gambiae s.l.* complex. Of 662 *An. gambiae s.l.* specimens analyzed, *An. coluzzii* represented 54.08% compared to 45.6% *An. gambiae* s.s. and 0.3% hybrid individuals (*An. gambiae* s.s./*An. coluzzii*) (Figure 2). However, no *An. arabiensis* or *An. melas* were identified. This could be explained by the fact that collections in the Atlantic Department were not made in a coastal lagoon area, where *An. melas* is found. In the departments of Littoral, Zou, Couffo and in the municipalities of Avrankou, Adja Ouèrè, Savè, Bopa and Karimama, *An. coluzzii* was the predominant species with frequencies ranging from 63.3% to 100%. By comparison, in the departments of Borgou, Donga, and Atacora and in the communes of Adjarra, Sakété, Ouèssè, Toffo, Houéyogbé and Pèrèrè, *An. gambiae* s.s was the major vector species (frequencies ranged between 51.7% and 93.3%), while in the commune of Zè, these two species cohabit (Figure 3).

Knock-down resistance (*kdr*) L1014F was the main resistance mechanism that had been observed in different departments for several years. In this study, *kdr* frequencies were highly heterogeneous between sites. In all surveyed departments, the proportion of RR genotypes was greater than or equal to 65%. The allelic frequencies [F (*kdr*)] were very high and varied from 68% in Borgou to 98% in Littoral, with an average of 87% at the national level. In departments such as Mono (95%), Zou and Donga (93%), Atacora (92%), Atlantique (90%) and Collines (88%), the frequencies were higher than average, whereas in Ouémé (84%), Plateau (81%), Alibori (80%) and Couffo (77%), the frequencies were lower than average.

The Ace-1 G119S mutation was also observed but at very low proportions with an allelic frequency [F (ace-1)] ≤ 5% in the surveyed departments. No RR genotype were observed; this mutation varied from 2% in Zou to 5% in Ouémé and Borgou, with an average of 3% in the country.

### 3.3. Anopheles Density and Biting Behavior by Commune and Department

After 768 nights of HLCs, a total of 7231 *Anopheles* were collected. The average human biting rate (HBR), estimated from the number of bites per man per night (b/m/n) was 9415 (Table 1) for all departments. Considering each department, the average HBRs were 34.594; 19.406; 15.094; 11.078; 9.219; 8.75; 6.906; 2.781; 1.562; 1.547; 1.125 and 0.922, respectively, in Littoral, Collines, Atacora, Alibori, Zou, Donga, Mono, Couffo, Borgou, Atlantic, Ouémé and Plateau. Significantly different HBRs between two communes of the same department were observed in all departments except Collines.

### 3.4. An. gambiae Sensu Lato Density and Biting Behavior Indoors and Outdoors

Table 2 shows the number of *An. gambiae* specimens collected during the study after two consecutive nights of capture in each commune from June to October 2017. Out of a total of 7231 *An. gambiae* collected, 3746 were collected indoors versus 3485 outside houses. Endophagy rate was 51.8%, i.e., 1 out of 2 *Anopheles* took bloodmeals from inside houses. The HBR of indoor *Anopheles* was very high in the departments of Littoral (34.41 b/m/n), Collines (18.5), Atacora (15.75), Alibori (11.1), Zou (10.19), Mono (9) and Donga (8.78). Similarly, HBRs were also quite high outside houses in Littoral (34.8 b/m/n), Collines (19.97), Atacora (14.44), Alibori (11.1), Zou (8.2) and Donga (8.72).

### 3.5. Sporozoite Index and Entomological Inoculation Rate (EIR) by Commune and by Department

A total of 2875 *An. gambiae s.l.* females were individually tested by CSP-ELISA, with 101 positive individuals detected; average infection index of 3.51%. The number of *An. gambiae* infective bites received per person per night is shown in Table 3. The EIR ranged from 0.016 in Ouémé and Plateau departments to 1.663 in Collines department.

### 3.6. Entomological Profile of Malaria Transmission across Benin

**Figure 4:**
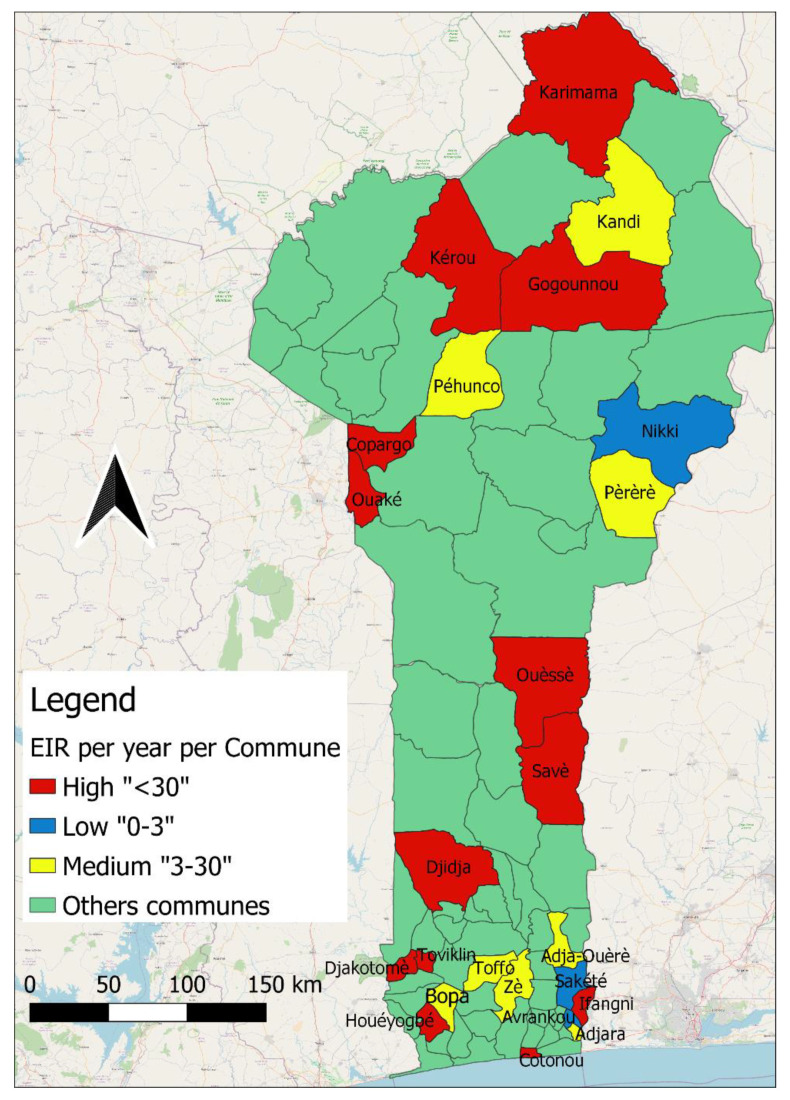
Map of the EIR (infectious bites per person per year) for sampled Communes.

Figure 4 shows the categorization of communes according to transmission intensity and shows the spatial distribution of malaria transmission across Benin. Accordingly, the communes of Avrankou, Sakété and Nikki were classified as low transmission (0 < EIR < 3 infectious bites/person/year), Adjarra, Adja, Ouèrè, Zè, Toffo, Bopa, Pehunco, Pèrèrè and Kandi were areas of medium transmission (3 < EIR < 30 infectious bites/person/year), while the remaining 12 communes were areas of high transmission (EIR > 30 infectious bites/person/year).

### 3.7. Parity rate of An. gambiae s.l., An. funestus Group and An. nili Group

**Figure 5:**
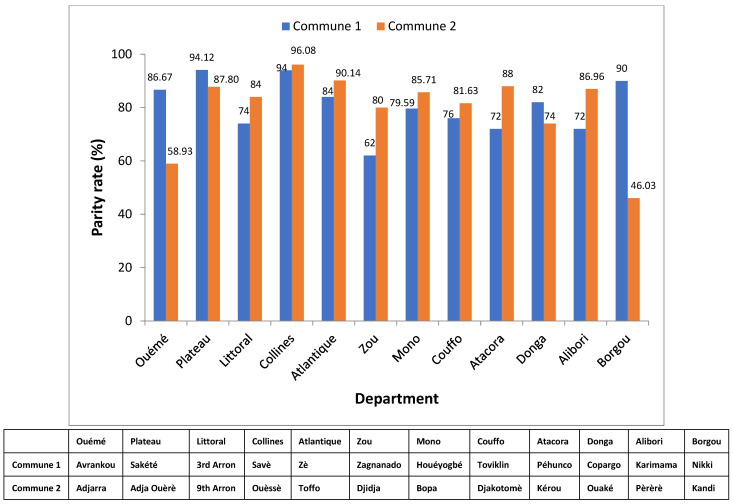
Parity rate of *An. gambiae s.l.*, *An. funestus* group and *An. nili* group.

The average parity rate was 78.6% (840 parous vectors out of 1069 dissected females) with moderate variation between departments. Parity rate was highest in Collines department (95.1%) (Figure 5) and lowest in Borgou department (52.1%; *p* < 0.05).

### 3.8. An. gambiae s.l Blood Meal Origins

Of 465 blood-fed *Anopheles* tested by ELISA, the majority took their blood meals from humans in all sites (92.04%; Table 4). In the communes of Savè, Djidja, Kérou, Karimama and Kandi, 6.67%; 6.67%; 2.79%; 6.67%; and 12.5% of *An. gambiae s.l.* took their blood meals from cows, respectively. By comparison, 9.1% of *An. gambiae s.l.* in Adja Ouèrè, 6.7% in Savè, Houéyogbé, Bopa and Kérou, 2.79% in Karimama, and 12.5% in Kandi fed on sheep. Blood meals from pigs occurred as follows for *An. gambiae s.l.*: 25% in Avrankou, 16.67% in Adjara, 6.67% in Houéyogbé and Bopa, and 13.33% in Djakotomè. It should be noted that in some communes, vectors took multiple blood meals from different animals and humans (Table 4).

### 3.9. Evaluation of the EIR According to Malaria Incidence and Prevalence in the Communes

**Figure 6:**
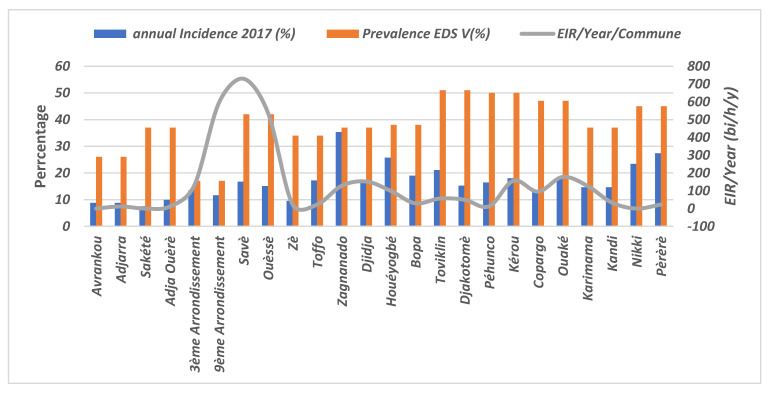
EIR, prevalence and incidence of malaria in different communes.

In general, the relationship between EIR, incidence and prevalence were not absolute across all communes (Figure 6). In some sites where the EIR was low, malaria incidence and prevalence were both high (e.g., Toviklin and Djakotome). In others, the EIR was very high, yet malaria incidence was medium and prevalence was high (e.g., Savè and Ouèssè); in Cotonou, EIR was high, but malaria incidence and prevalence were both low. In Karimama, Copargo, Ouaké, Kérou and Pèrèrè in the north of the country; in Zangnanado and Djidja in Zou; and in the departments of Mono and Couffo, all three metrics were consistent with one another.

Correlation tests between the EIR and malaria incidence and prevalence were not significant. Communes with high levels of entomological transmission did not show significant associations with incidence and prevalence rates.

## 4. Discussion

Knowledge of the pattern of malaria transmission has proven to be a prerequisite not only for understanding the epidemiology of the disease but also for deploying effective and targeted strategies to control mosquito vectors [10]. This study revealed substantial geographical differences in entomological indicators of malaria transmission in Benin. Malaria transmission was diverse across the country, with certain communes identified, which require special attention for planned vector control interventions. A large proportion of older vectors (i.e., potentially infective vectors) observed in many areas, with sparse pre-existing data, emphasized that additional control measures must be deployed in these localities.

Study findings confirmed that *An. gambiae s.l.* (97.8%) and *An. funestus* (1.1%; present in 7 departments out of 12 surveyed) were the main vector species groups, consistent with others studies conducted in the country [18,44,45,46,47,48]. The low abundance of *An. funestus* may be due to the absence of its typical larval habitat (permanent or semi-permanent shaded freshwater streams, swamps, ponds and lakes) in these areas. In most sites, almost equal proportions of members of the *An. gambiae s.l.* complex were observed sympatrically (*An. coluzzii*: 54.08% and *An. gambiae* s.s.: 45.6%) [48]. Furthermore, *An. nili* (1.1%) was identified as an additional vector species in the commune of Kérou, Atacora department, in line with previous reports [11]. This study did not collect any *An. arabiensis*, a vector of dry and limited savannah areas, which has recently been found in Benin in the communes of Malanville, Parakou Dassa and Allada [18]. This likely reflects ecological features of the sampling locations selected for this cross-sectional study. Similarly, no brackish water areas were surveyed and thus no *An. melas* were collected either [49]. Spatial variation in *An. gambiae s.l.* and other vector species densities could be explained by the relative ecological characteristics of each site and their propensity to support vector breeding; many agricultural plains, rice, cotton and vegetable growing areas, such as Karimama, Kérou, Savè and Ouèssè are highly suitable for vector breeding [18,50,51]. In other endemic regions, such as Zitta, Côte d’Ivoire [38], high EIRs have been associated with rice, vegetable and cotton cultivation areas [42], which may explain the intense transmission observed in Savè (729.72 infective bites/human/year), Ouèssè (537.84), Kérou (156.6), Djidja (151.92), Karimama (128.16) and Zangnando (127.8).

Regarding vector behavior, *An. gambiae s.l*. densities were largely comparable inside and outside houses. This result is similar to that observed by Gnaguenon et al. [18], demonstrating that in areas that have not received IRS, vectors displayed low levels of endophagy. Spatial heterogeneity in EIRs was evidenced by infectious vector bites being more common in rural areas compared to urban parts of the same district. Together study results highlight the importance of understanding micro-ecological conditions and environmental characteristics, which in turn determine the intensity of malaria transmission. In total, 12 out of 24 communes were characterized by the highest EIRs, namely, Copargo, Cotonou, Djakotome, Djidja, Houeyogbe, Ifangni, Karimama, Kerou, Ouake, Ouesse, Save, and Toviklin, with more than 8 infectious bites/man/night suggesting that the populations of Cotonou, Savè, Ouesse, Djidja, Kerou, Ouaké Copargo, and Karimama were more exposed to malaria transmission, compared to other localities. For Karimama, sporozoite rates were low, implicating high levels of vector biting, linked to the permanent availability of breeding sites in rice cultivation areas, as was the case a few years ago in Malanville [18]. Similarly, in Cotonou, optimal conditions for vector breeding have been described in urban areas, with 593.64 infective bites per human per night recorded in the 9th arrondissement, compared to 130 in the 3rd arrondissement. These areas often feature high levels of nuisance biting by other culicines, breeding in polluted water [52,53]. Parallel observations have been reported from East Africa and from Dar es Salaam, Tanzania [53,54], Ouagadougou, Burkina Faso [55], Tori-Bossito, southern Benin [56], Kandi, northeastern Benin [57], northwestern South Africa (Kazembe et al. 2005) and southern Benin [18]. Furthermore, the proportion of *An. gambiae s.l.* that tested positive for *P. falciparum* was very high in the Collines department (10.0%), reinforcing the need to implement supplemental vector control strategies in this region of the country that benefits only from conventional interventions from the NMCP. A similar control strategy is underway in some communes of Atacora and Alibori in northern Benin that already receive IRS and SMC.

Other factors that also likely contributed to differences in EIRs included increased vector contact, facilitated by human behaviors (e.g., late bedtimes, sleeping outside during the hot dry season and non-use of LLINs, etc.), those which adversely impacted LLIN physical integrity (e.g., use of sharps and lit candles) and bioefficacy (e.g., high frequency of washing LLINs). These observations underscore the need to support malaria vector control initiatives with appropriate information, education, and communication strategies to ensure effective intervention use. The impact of currently deployed LLINs on vector populations may also explain the low vector densities and transmission risk in some localities. Interestingly, malaria prevalence data, collected during the same study period was not correlated with EIRs [25,58] nor with malaria incidences in the population in the same year. This has been documented by other authors [59,60,61] with the most important contextual factor incriminated being the degree of malaria seasonality in the areas assessed. Further work is warranted to characterize the patterns of *P. falciparum* transmission by mosquito vectors to inform the implementation of insecticide-based vector control interventions in Benin, where several large-scale vector control interventions (LLINs and IRS) are underway. This information will guide policies in the selection of priority areas for primary and secondary vector control interventions in line with the move towards elimination [62,63].

Regarding the presence of resistance mechanisms in *An. gambiae s.l.*, the L1014F kdr mutation was found at very high frequencies in its twin species without any differences (*An. gambiae* s.s. and *An. coluzzii*) in the country, similar to previous work carried out in Benin of Gnanguenon et al. [64], Yahouédo et al. [65], Salako et al. [44] and Akogbeto et al. [48]. The trend was the same for the relatively low allelic frequencies of the G119S Ace-1 mutation mechanism of *An. gambiae* and *An. coluzzii* [11,48,64].

## 5. Conclusions

The study demonstrated the heterogeneous and diverse nature of malaria transmission in Benin. Spatial variation in the behavior and distribution of malaria vectors was observed across the country. The study also highlighted differences in human exposure to infectious bites of malaria vectors during periods of high transmission and that entire communes are without transmission data but benefit from interventions. Communes in the country such as Savè, Ouèssè, Djidja and Houéyogbé deserve even more attention in order to better protect the populations most at-risk. The profile of malaria transmission in the country is a crucial piece of information and this should be updated every year to identify areas for targeted intervention deployment; strengthening communication strategies for social and behavioral change is also crucial for effective malaria vector control. Challenges related to vector resistance and insufficient transmission information remain.

## Figures and Tables

**Table 1:** Bite rate per man per night (HBR) by commune and department.

Department	Communes (Latitude and Longitude)	Total Collected	Man Night	HBR/Commune	P1	HBR/Department
Ouémé	Avrankou (6°33′32.07″ N 2°38′56.93″ E)	14	32	0.438	<0.001	1.125 ^a^
	Adjarra (6°31′22.69″ N 2°39′46.03″ E)	58	32	1.813		
Plateau	Sakété (6°44′12.47″ N 2°39′49.01″ E)	18	32	0.563	0.003	0.922 ^a^
	Adja Ouèrè (6°57′13.25″ N 2°34′44.25″ E)	41	32	1.281		
Littoral	3ème Arrond (6°22′12.76″ N 2°23′28.43″ E)	964	32	30.125	<0.001	34.594 ^b^
	9ème Arrond (6°23′24.15″ N 2°24′03.44″ E)	1250	32	39.063		
Collines	Savè (8°01′59.46″ N 2°29′08.17″ E)	627	32	19.594	0.78	19.406 ^c^
	Ouèssè (8°29′35.07″ N 2°25′25.42″ E)	615	32	19.219		
Atlantique	Zè (6°42′06.88″ N 2°20′31.84″ E)	26	32	0.813	<0.001	1.547 ^d^
	Toffo (6°50′24.08″ N 2°04′45.67″ E)	73	32	2.281		
Zou	Zagnanado (7°16′47.86″ N 2°24′14.50″ E)	149	32	4.656	0	9.219 ^e^
	Djidja (7°20′45.80″ N 1°56′08.19″ E)	441	32	13.781		
Mono	Houéyogbé (6°31′52.28″ N 1°52′15.52″ E)	273	32	8.531	<0.001	6.906 ^f^
	Bopa (6°42′04.67″ N 1°57′01.73″ E)	169	32	5.281		
Couffo	Toviklin (6°56′24.12″ N 1°46′23.30″ E)	73	32	2.28	0.016	2.781 ^g^
	Djakotomè (6°53′45.68″ N 1°41′47.51″ E)	105	32	3.281		
Atacora	Péhunco (10°30′02.35″ N 2°13′56.68″ E)	128	32	4	0	15.094 ^h^
	Kérou (10°49′51.82″ N 2°06′38.25″ E)	838	32	26.188		
Donga	Copargo (9°50′22.22″ N 1°32′51.32″ E)	254	32	7.938	0.028	8.75 ^e^
	Ouaké (9°39′47.81″ N 1°23′12.90″ E)	306	32	9.563		
Alibori	Karimama (12°04′08.91″ N 3°11′02.00″ E)	684	32	21.375	0	11.078 ^h^
	Kandi (11°07′51.02″ N 2°56′05.75″ E)	90	32	2.813		
Borgou	Nikki (9°55′51.14″ N 3°12′36.40″ E)	10	32	0.313	0	1.562 ^d^
	Pèrèrè (9°47′31.29″ N 2°59′26.45″ E)	25	32	0.781		
Total		7231	768	9.415		

P1: *p*-value of the test rate ratio comparing HBRs between two communes of the same department; per column, the HBRs affected by the same letter do not differ significantly (*p* > 0.05).

**Table 2:** Indoor versus outdoor biting rates by commune and department.

Departments	Communes	Indoor Total	Outdoor Total	HBR Indoor	HBR Outdoor	P1
Ouémé	Avrankou	9	5	0.563	0.313	0.285
	Adjarra	32	26	2	1.625	0.431
Plateau	Sakété	13	5	0.813	0.313	0.059
	Adja Ouèrè	23	18	1.438	1.125	0.435
Littoral	3ème Arrondissment	469	495	29.313	30.938	0.402
	9ème Arrondissment	632	618	39.5	38.625	0.692
Collines	Savè	256	371	16	23.188	<0.001
	Ouèssè	347	268	21.688	16.75	0.001
Atlantique	Zè	20	6	1.25	0.375	0.006
	Toffo	50	23	3.125	1.438	0.002
Zou	Zagnanado	79	70	4.938	4.375	0.461
	Djidja	247	194	15.438	12.125	0.012
Mono	Houéyogbé	174	99	10.875	6.188	<0.001
	Bopa	114	55	7.125	3.438	<0.001
Couffo	Toviklin	48	25	3	1.563	0.007
	Djakotomè	65	40	4.063	2.5	0.019
Atacora	Péhunco	74	54	4.625	3.375	0.077
	Kérou	430	408	26.875	25.5	0.447
Donga	Copargo	122	132	7.625	8.25	0.53
	Ouaké	159	147	9.938	9.188	0.687
Alibori	Karimama	342	342	21.375	21.375	1
	Kandi	23	67	1.438	4.188	<0.001
Borgou	Nikki	5	5	0.313	0.313	1
	Pèrèrè	13	12	0.813	0.75	0.841
Total		3746	3485	11.706	10.891	

P1: *p*-value of the test rate ratio comparing indoor HBR to outdoor HBR in each commune.

**Table 3:** Sporozoite Index and EIR by commune and department.

Departement	Commune	Total Tested	N Positif	SI	HBR	Commune EIR	EIR 95%CI	EIR	EIR/Year/
							Commune	Department	Department
Ouémé	Avrankou	14	0	0	0.44	0	0–0.008	0.016 ^a^	5.7
	Adjarra	57	1	0.018	1.81	0.032	0.024–0.041		
Plateau	Sakété	18	0	0	0.57	0	0–0.006	0.016 ^a^	5.63
	Adja Ouèrè	41	1	0.024	1.29	0.031	0.022–0.042		
Littoral	3^ème^ Arrondissment	250	3	0.012	30.13	0.362	0.348–0.375	0.992 ^b^	357.1
	9^ème^ Arrondissment	308	13	0.042	39.1	1.649	1.623–1.674		
Collines	Savè	87	9	0.103	19.6	2.027	1.974–2.081	1.663 ^c^	598.82
	Ouèssè	193	15	0.078	19.2	1.494	1.463–1.524		
Atlantique	Zè	25	2	0.08	0.81	0.065	0.049–0.085	0.063 ^d^	22.73
	Toffo	73	2	0.027	2.28	0.062	0.053–0.073		
Zou	Zagnanado	105	8	0.076	4.66	0.355	0.335–0.375	0.403 ^e^	145.08
	Djidja	261	8	0.031	13.78	0.422	0.409–0.437		
Mono	Houéyogbé	217	7	0.032	8.53	0.275	0.263–0.288	0.18 ^f^	64.67
	Bopa	129	2	0.016	5.28	0.082	0.073–0.091		
Couffo	Toviklin	73	5	0.068	2.28	0.156	0.141–0.173	0.153 ^g^	55.24
	Djakotomè	72	3	0.042	3.28	0.137	0.122–0.153		
Atacora	Péhunco	116	1	0.009	4	0.034	0.029–0.041	0.211 ^h^	76.1
	Kérou	241	4	0.017	26.19	0.435	0.42–0.45		
Donga	Copargo	60	2	0.033	7.94	0.265	0.242–0.289	0.39 ^e^	140.45
	Ouaké	97	5	0.052	9.57	0.493	0.469–0.518		
Alibori	Karimama	300	5	0.017	21.37	0.356	0.344–0.368	0.238 ^i^	85.63
	Kandi	90	3	0.092	2.81	0.094	0.046–0.083		
Borgou	Nikki	10	0	0	0.31	0	−0.106	0.042 ^j^	15.07
	Pèrèrè	25	2	0.077	0.78	0.062	0.082–0.012		
Total		2875	101	0.0351	9.42	0.331			

N = positive number, SI = Sporozoite Index; EIR = Entomological Inoculation Rate; CI = Confidence Interval. Per column, the EIRs affected by the same letter do not differ significantly (*p* > 0.05).

**Table 4:** Origins of blood meals of *An. gambiae s.l.* females collected in the departments of Benin.

Department	Commune	Total Collected	Blood Meal Origins			
			N Human (%)	N Cow (%)	N Sheep (%)	N Pig (%)
Ouémé	Avrankou	4	3 (75)			1 (25)
	Adjarra	6	5 (83.33)			1 (16.67)
Plateau	Sakété	1	1 (100)			
	Adja Ouèrè	11	11 (100)		1 (9.1)	
Littoral	3^ème^ Arrondissment	29	29 (100)			
	9^ème^ Arrondissment	45	45 (100)			
Collines	Savè	45	39 (86.67)	3 (6.67)	3 (6.67)	
	Ouèssè	20	20 (100)			
Atlantique	Zè	3	3 (100)			
	Toffo	1	1 (100)			
Zou	Zagnanado	14	14 (100)			
	Djidja	45	45 (100)	3 (6.67)		
Mono	Houéyogbé	45	42 (93.33)		3 (6.67)	3 (6.67)
	Bopa	15	14 (93.33)		1 (6.67)	1 (6.67)
Couffo	Toviklin	20	20 (100)			
	Djakotomè	15	15 (100)			2 (13.33)
Atacora	Péhunco	12	12 (100)			
	Kérou	15	10 (66.67)	1 (2.79)	1 (6.67)	
Donga	Copargo	20	20 (100)			
	Ouaké	15	12 (100)			
Alibori	Karimama	75	60 (80)	5 (6.67)	5 (2.79)	
	Kandi	8	6 (75)	1 (12.5)	1 (12.5)	
Borgou	Nikki	0				
	Pèrèrè	1	1 (100)			
Total		465	428 (92.04)	13 (2.79)	15 (3.22)	8 (1.72)

## Data Availability

Data is contained within the article.
